# Supplementary material for: GmWAK1, Novel Wall-Associated Protein Kinase, Positively Regulates Response of Soybean to Phytophthora sojae Infection
Source: Int J Mol Sci. 2023 Jan 2;24(1):798. doi: 10.3390/ijms24010798 (PMC9821614; doi:10.3390/ijms24010798)
Supplement: Supplementary file 1 [file ijms-24-00798-s001.zip › ijms-2122624-supplementary.pdf]

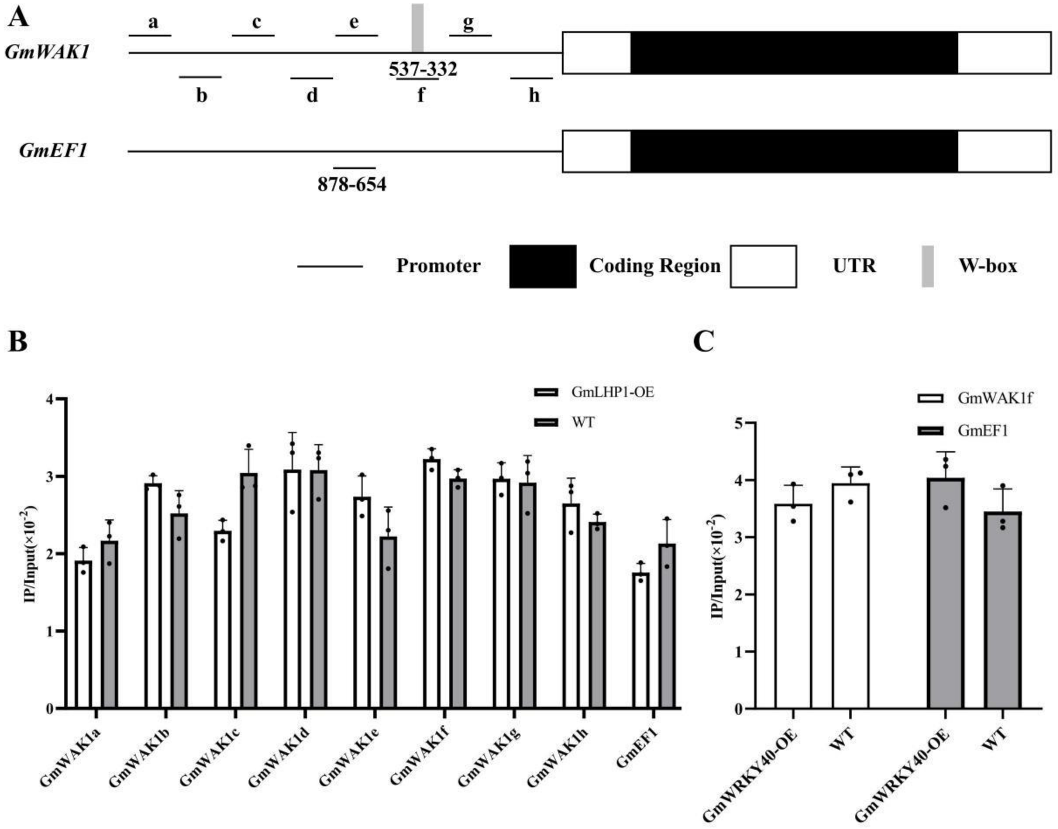


**Figure S1.(A)** The GmWAK1 promoter region is divided into a-h eight segments, where f contains W-box elements, pGmEF1 was used as a negative control. **(B, C)** The broken chromatin precipitation from the specific primers targeting the GmWAK1 promoter region was analyzed by qPCR.Three biological replicates, each with three technical replicates, were averaged and statistically analyzed using Student's t-test (*P < 0.05, **P < 0.01). Bars indicate standard deviation of the mean (n = 3)

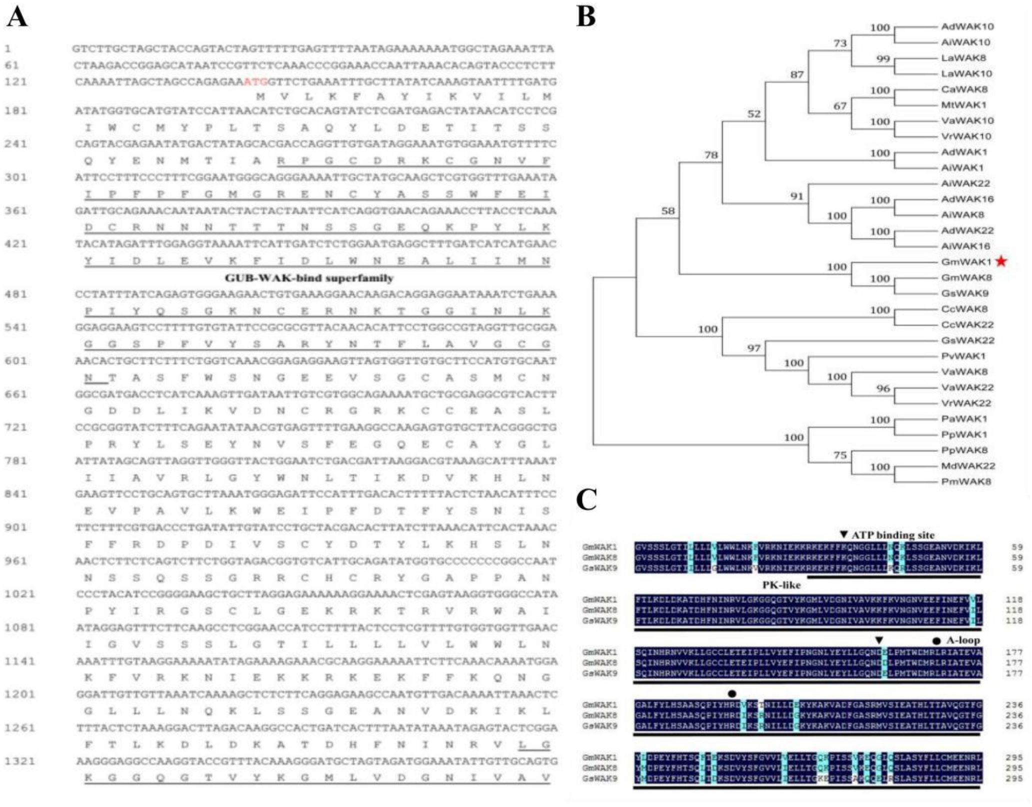


**Figure S2. Sequence and structure of GmWAK1. (A)** Nucleotide and amino acid sequence of *GmWAK1*. The GUB-WAK-bind superfamily motif and PKc-like superfamily motif are marked by underlined. **(B)** Phylogenetic analysis of GmWAK1 and protein of other plant species. The GenBank Accession numbers are as follows: AdWAK1(XP_015959308.1),AdWAK10(XP_020995693.1),AdWAK16(XP_015959372.1),AdWAK22(XP_015959331.1),AiWAK1(XP_020976395.1),AiWAK8(XP_016197846.1),AiWAK10(XP_020976394.1),AiWAK16(XP_016195123.2),AiWAK22(XP_016197847.1),CaWAK8(XP_004486489.1),CcWAK8(XP_020230764.1),CcWAK22(KYP52032.1),GmWAK1(XP_003534786.2),GmWAK8(XP_003534785.2),GsWAK9(KHN44651.1),GsWAK22(KHN40782.1),LaWAK8(XP_019418697.1),LaWAK10(XP_019425178.1),MdWAK22(XP_008380329.1),MtWAK1(XP_003594613.1),PaWA

K1(XP_021811291.1),PmWAK8(XP_016648027.1),PpWAK1(XP_020420546.1),PpWAK8(XP_020414680.1),PvWAK1(XP_007147589.1),VaWAK8(XP_017434413.1), VaWAK10(XP_017434744.1),VaWAK22(XP_017434391.1),VrWAK10(XP_022642565.1),VrWAK22(XP_022642974.1). **(C)** Alignment of amino acid sequences of GmWAK1 and the nearby 2 WAK proteins.

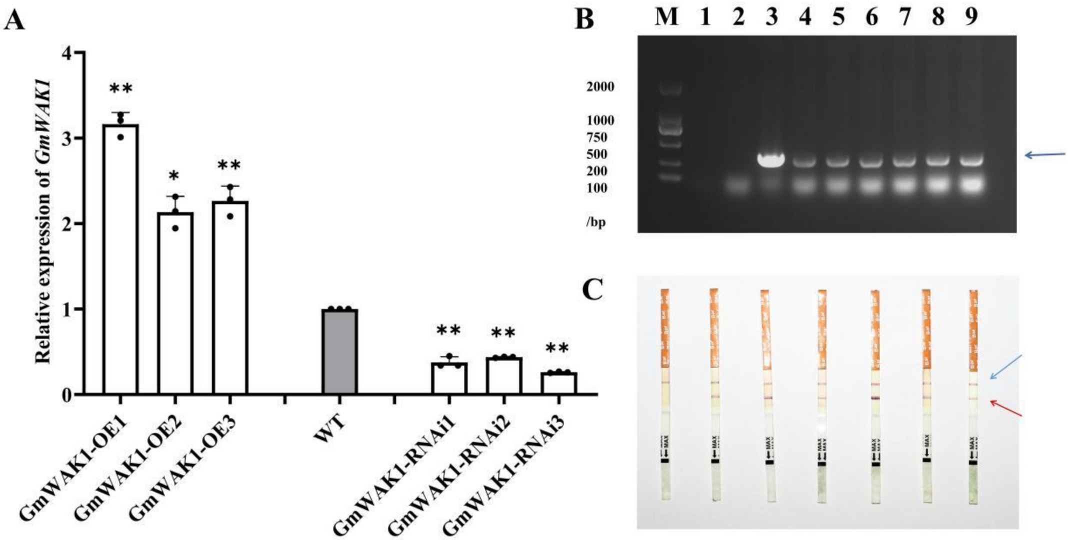


**Figure S3.** **(A)** GmWAK1 gene expression level in transgenic and WT soybean by qRT-PCR. Significant differences were analyzed based on the results of three biological replications (Student's t test: ** $p < 0.01$). Bars indicate the standard error of the means. **(B)** gel image of PCR products obtained with primer sets for T-DNA regions of the vector. M: DL2000 marker. 1: negative control (dd$H_2O$). 2: DNA of WT soybean plants. 3: plasmid of the pCAMBIA3301-GmWAK1 vector. 4 - 6: GmWAK1-OE1,2,3. 7-9: GmWAK1-RNAi1,2,3. **(C)** Detection of the selectable marker gene bar by test strip. Blue arrow: control. Red arrow: bar is positive.

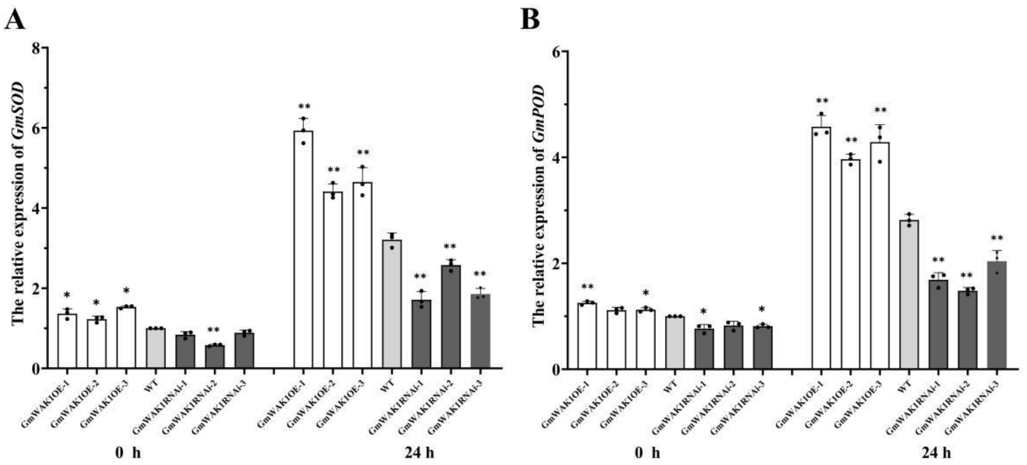


**Figure S4. Analysis of antioxidant enzyme relative expression of genes under mock treatment and infected by *Phytophthora sojae* at 24 h post-inoculation (hpi).** The experiment was performed on three biological replicates, each with three technical replicates, and statistically analysed using Student's t-test (*P < 0.05, **P < 0.01). Bars indicate the standard error of the mean.

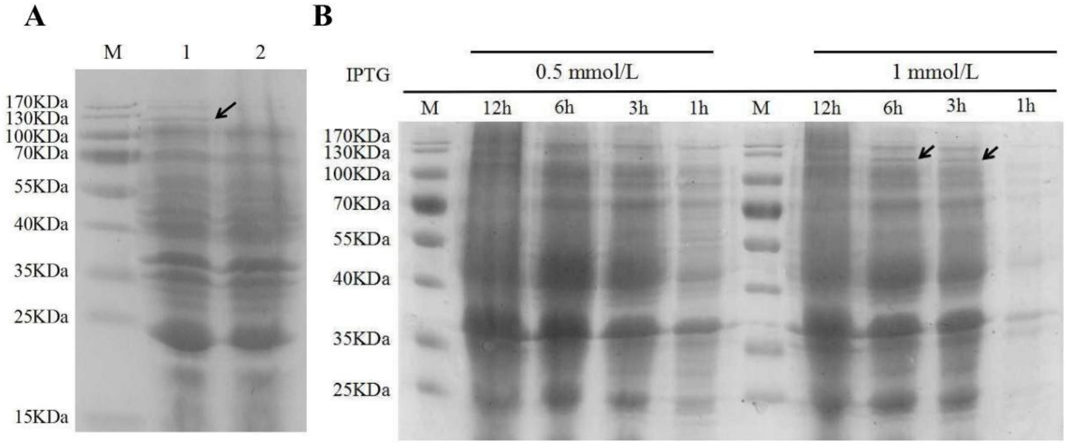


**Figure S5. GmWAK1 fusion protein. (A)** Expression of recombinant GmDIR22 protein in *E.coli* BL21 (DE3) was induced using 1.0 mM IPTG at 37 °C for 4 h. Lane 1 pET29b(+)-GmWAK1 induced by IPTG for 4 h, Lane 2 pET-29b(+) vector induced

by IPTG for 4 h. **(B)** Expression of recombinant GmWAK1-His protein in different IPTG concentration induction.

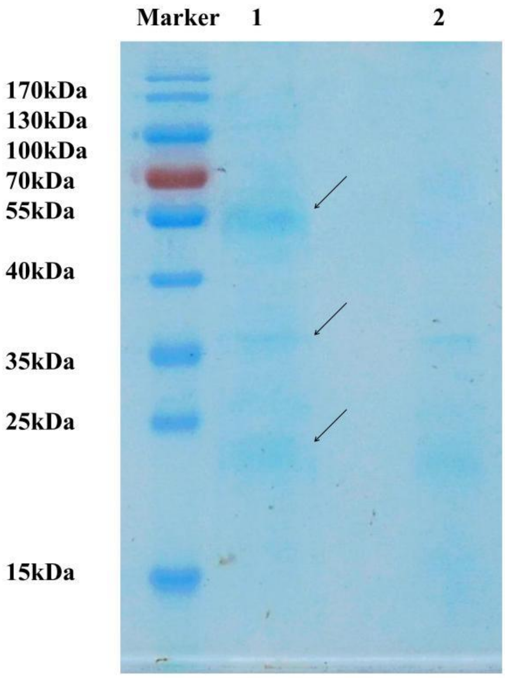


**Figure S6. The protein interacting with GmWAK1 was screened by immunoprecipitation from soybean total protein.**

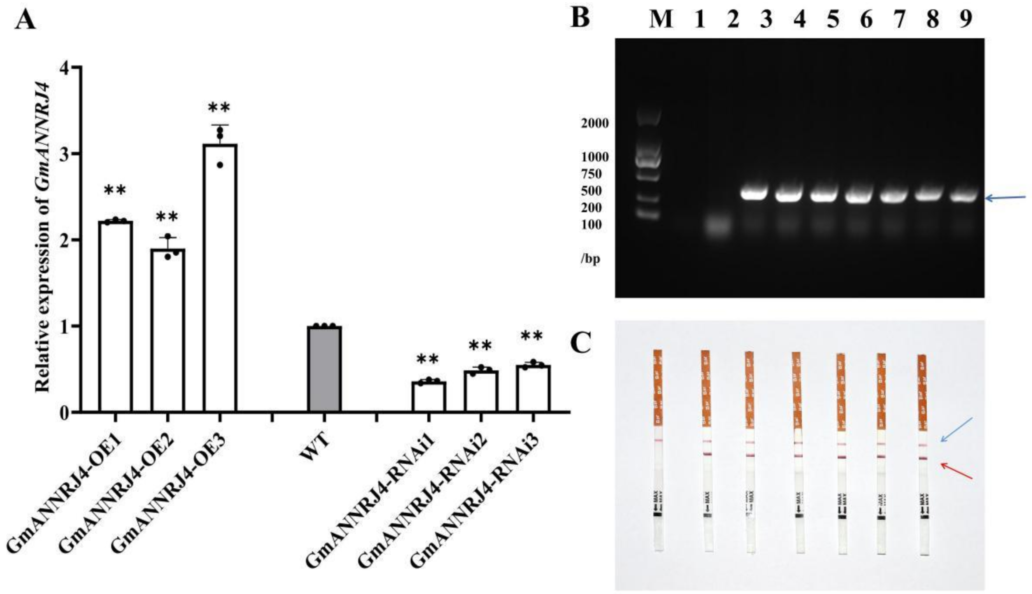


**Figure S7. (A)** GmANNRJ4 gene expression level in transgenic and WT soybean by qRT-PCR. Significant differences were analyzed based on the results of three biological replications (Student's t test: ** $p < 0.01$). Bars indicate the standard error of the means. **(B)** gel image of PCR products obtained with primer sets for T-DNA regions of the vector. M: DL2000 marker. 1: negative control ($ddH_2O$). 2: DNA of WT soybean plants. 3: plasmid of the pCAMBIA3301-GmANNRJ4 vector. 4 – 6: GmANNRJ4-OE1,2,3. 7-9: GmANNRJ4-RNAi1,2,3. **(C)** Detection of the selectable marker gene bar by test strip. Blue arrow: control. Red arrow: bar is positive.

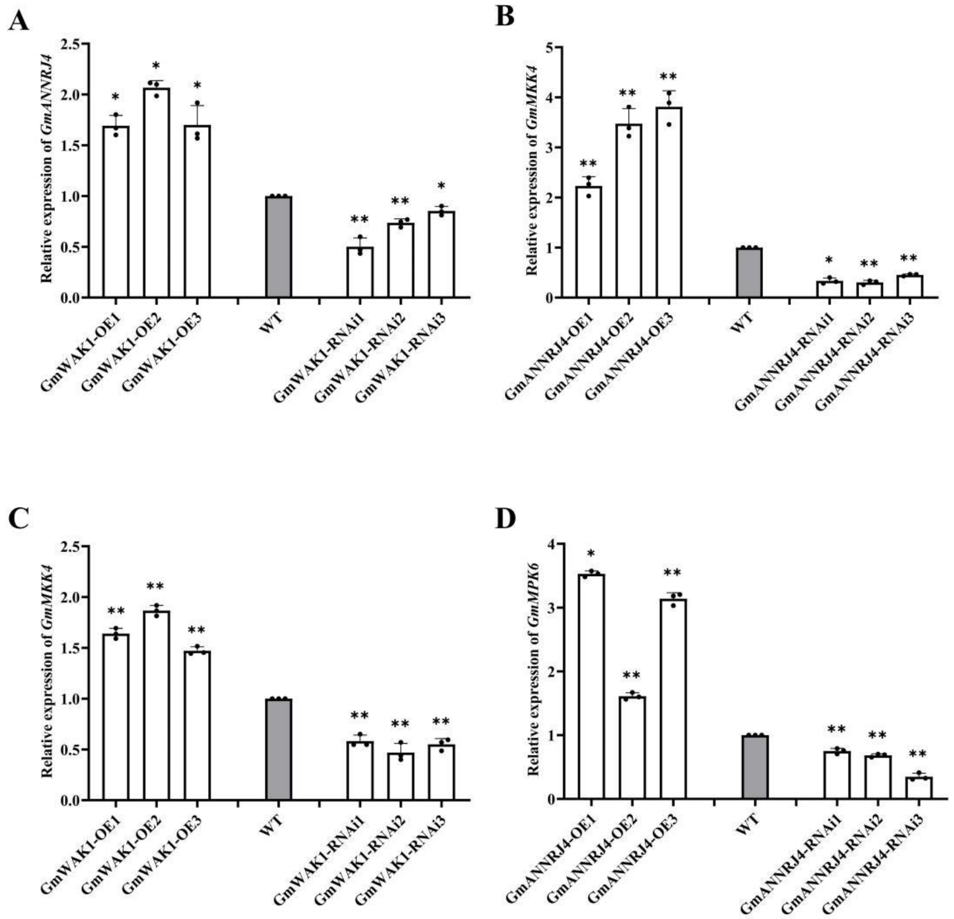


**Figure S8. (A, C)** Relative expression levels of GmANNRJ4 and GmMKK4 in GmWAK1 transgenic soybean. **(B, D)** Relative expression levels of GmMKK4 and GmMPK6 in GmANNRJ4 transgenic soybean. The experiment was performed on three biological replicates, each with three technical replicates, and was statistically analysed using Student's t-test (*P < 0.05, **P < 0.01). Bars indicate the standard error of the mean.

**Supplemental Table S1. List of primers used in this study.**

**Primers for constructs in plant transformation**

| Primer name | Primers（5'→3'） |
|---|---|
| *GmANNRJ4-Ox*F | 5' GAATTCATGGCTACCCTTGTTGCTCCAA 3' |
| *GmANNRJ4-Ox*R | 5' TCTAGAATCTTCCTTCCCCAACAGAGTG 3' |
| *GmWAK1-Ox*F | 5' GAGATCTAAATGGTTCTGAAATTTGCTTATATC 3' |
| *GmWAK1-Ox*R | 5' GCACGTGCCATGATTAGGATGAGTAGGCATAAT 3' |
| *GmWAK1-RNAi1*F | 5' CTCGAGCTGGTCAAACGGAGAGGAAGT 3' |
| *GmWAK1-RNAi1*R | 5' CCATGGATAATCAGCCCGTAAGCACACT 3' |
| *GmWAK1-RNAi2*F | 5' TCTAGACTGGTCAAACGGAGAGGAAGT 3' |
| *GmWAK1-RNAi2*R | 5' GGATCC ATAATCAGCCCGTAAGCACACT 3' |
| *GmANNRJ4-RNAi1*F | 5' CTCGAGCTTGTTGCTCCAATCACCTTT 3' |
| *GmANNRJ4-RNAi1*R | 5' CCATGGTCATAAACCTTTCTGATTTGTT 3' |
| *GmANNRJ4-RNAi2*F | 5' TCTAGACTTGTTGCTCCAATCACCTTT 3' |
| *GmANNRJ4-RNAi2*R | 5' GGATCCTCATAAACCTTTCTGATTTGTT 3' |
| *Bar*-R | 5' GGTCTGCACCATCGTCAACCACT 3 |
| *Bar*-F | 5' ATATCCGAGCGCCTCGTGCAT 3' |

**Primers for quantitative real-time PCR**:

| Primer name | Primers（5'→3'） |
|---|---|
| *GmPR3*F | 5' AACACCAACTCCAACAACACCTA 3' |
| *GmPR3*R | 5' TGCCAAAGCCATTGAAAGA 3' |
| *GmPR1*F | 5' GGCCAATACGGGGAGAATCT 3' |
| *GmPR1*R | 5' TCCAAACAACCTGAGTGTAATGC 3' |
| *GmPR10*F | 5' AACATCTTCTCACAACAGCATCAC 3' |
| *GmPR10*R | 5' AGGCACAGCAGCAGGGGTAT 3' |
| *GmPR2*F | 5' GTTCGGAATGTGAAGCAAGGA 3' |
| *GmPR2*R | 5' ATAGGAGAAAAGAGCCGCCAA 3' |
| *GmMPK6*F | 5' GCATCGTTTGCTCGGCTTT 3' |
| *GmMPK6*R | 5' TCTCCCTTTGAGGTGGTGG 3' |
| *GmMKK4*F | 5' AGGGTCACTGGAGGGGAAAC 3' |
| *GmMKK4*R | 5' CGCGTAAGCGTCGTATTGC 3' |
| *EF1β*F | 5' CCACTGCTGAAGAAGATGATGATG 3' |
| *EF1β*R | 5' AAGGACAGAAGACTTGCCACTC 3' |
| *GAPHD*F | 5' CCAGTAAGGATGCCCCCATG 3' |
| *GAPHD*R | 5' CAAGGCAGTTGGTTGTGCAG 3' |
| *TEF1*F | 5' TGATCGTGCTGAACCACCC 3' |
| *TEF1*R | 5' CGAGCGACGGTCCATCTT 3' |
| *GmWAK1*F | 5' TTGTATCCTGCTACGACACTTATCT 3' |
| *GmWAK1*R | 5' ACTCCTATTATGGCCCACCTT 3' |
| *GmPAL-Q*F | 5' GCAGTGACTGGGTGATGG 3' |

| | |
|---|---|
| *GmPAL-Q*R | 5' GCTCTGGTTGCTGTGTGG 3' |
| *GmSOD1*-QF | 5' CAGTTCTTGGCAGCAGCGA 3' |
| *GmSOD1*-QR | 5' CACCGTGCTCGTTGTTATTAGG 3' |
| *GmPOD*-QF | 5' AAGCCTCATTCGCCTCCAC 3' |
| *GmPOD*-QR | 5' CAAGATACGACTCCAGGGCAA 3' |
| *GmANNRJ4-QF* | 5' GTTCATCAGAGGCAACAAATCA 3' |
| *GmANNRJ4-QR* | 5' TTAGTAGCCACATCTTCTTCCAAA 3' |

**Primers for constructs in BiFC assays**

| Primer name | Primers（5'→3'） |
|---|---|
| *GmWAK1-nYFP*F | 5' CCATGGAAATGGTTCTGAAATTTGCTTATATC 3' |
| *GmWAK1-nYFP*R | 5' GGATCCCATGATTAGGATGAGTAGGCATAAT 3' |
| *GmANNRJ4-cYFP*F | 5' CTCGAGAATGGCTACCCTTGTTGCTCCAA 3' |
| *GmANNRJ4-cYFP*R | 5' GAATTCATCTTCCTTCCCCAACAGAGTGAG 3' |
| *GmPIP2-cYFP*F | 5' CTCGAGAATGGCCAAAGACGTTGAGCAGG 3' |
| *GmPIP2-cYFP*R | 5' GAATTCAGTGTTGCTCCTGAATGATCCAAG 3' |
| *GmOEE3-cYFP*F | 5' CTCGAGAATGGCTCAAGCAATGGCATCAA 3' |
| *GmOEE3-cYFP*R | 5' GAATTCACCAAGTTTGGCAAGGACATCGTT 3' |

**Primers for constructs in pull-down assays**

| Primer name | Primers（5'→3'） |
|---|---|
| *GmANNRJ4-GST*F | 5' GAATTCATGGCTACCCTTGTTGCTCCAA 3' |
| *GmANNRJ4-GST*R | 5' GTCGACTCAATCTTCCTTCCCCAACAGA 3' |
| *GmWAK1-His*F | 5' CCATGGAAATGGTTCTGAAATTTGCTTATATC 3' |
| *GmWAK1-His*R | 5' AAGCTTATGATTAGGATGAGTAGGCATAAT 3' |

**Primers for constructs in Subcellular Lcalization assays**

| Primer name | Primers（5'→3'） |
|---|---|
| *GmWAK1*GFP-F | 5' GGATCCAATTACTAAGACCGGAGCATA 3' |
| *GmWAK1*GFP-R | 5' AAGCTTTTTTACACACAATACAAACAAGT 3' |

**Primers for constructs in ChIP assays**

| Primer name | Primers（5'→3'） |
|---|---|
| *GmWAK1a*-F | 5' GGGAATTTTCGTGCGACTATG 3' |
| *GmWAK1a*-R | 5' ATGGACTTTATGACCAATGAACTTT 3' |
| *GmWAK1b*-F | 5' CAAAAAAGGAAAGTTCATTGGTCA 3' |
| *GmWAK1b*-R | 5' TTATTTAGTAAATTATGCCGAAGCATT 3' |
| *GmWAK1c*-F | 5' CCTCTAATCATGTTCGGCACC 3' |
| *GmWAK1c*-R | 5' CAATCAAGGAACTCATGCTTTTATAC 3' |
| *GmWAK1d*-F | 5' CATGAGTTCCTTGATTGTTTGA 3' |

| | |
|---|---|
| *GmWAK1d*-R | 5' TGTCTGGCAGGGGATTCTT 3' |
| *GmWAK1e*-F | 5' AGAATCCCCTGCCAGACAA 3' |
| *GmWAK1e*-R | 5' TAATGGTACATGCGACGTATCAT 3' |
| *GmWAK1f*-F | 5' TGGCCACATGCCGACAT 3' |
| *GmWAK1f*-R | 5' GCGTGCAACCTACCCTCA 3' |
| *GmWAK1g*-F | 5' CTAAAGTCAGGCTAAGGTTGTTG 3' |
| *GmWAK1g*-R | 5' GCCGGATATTTCTATATATATGCT 3' |
| *GmWAK1h*-F | 5' ATCTTTTGTTCAAGCACAGGACC 3' |
| *GmWAK1h*-R | 5' CGCACGTTAGAATAACTCACCG 3' |
| *GmEF1*-aF | 5' TCGGGTCAGATGTCGGATG 3' |
| *GmEF1*-aR | 5' TACAGTGCGAGCGAGCGT 3' |

**Supplemental Table S2 Part sequencing results of GmWAK1 associated proteins by LC-MS/MS analysis**

| No. | Gene Bank accession no. | Gene Description |
|---|---|---|
| C-W-1 | tr\|C6SZS9\|C6SZS9 | Dirigent protein |
| C-W-2 | tr\|Q2HZ34\|Q2HZ34 | Plasma membrane-associated AAA-ATPase |
| C-W-3 | tr\|Q43453\|Q43453 | G.max mRNA from stress-induced gene (H4) |
| C-W-4 | tr\|Q9SE03\|Q9SE03 | Copper chaperone homolog CCH |
| C-W-5 | tr\|I1JKS0\|I1JKS0 | Pectinesterase |
| C-W-6 | tr\|Q38JE0\|Q38JE0 | Temperature-induced lipocalin |
| C-W-7 | tr\|F6KBT4\|F6KBT4 | Allene oxide cyclase 4 |
| C-W-8 | tr\|I1M0U7\|I1M0U7 | Annexin |
| C-W-9 | tr\|D6C4Z9\|D6C4Z9 | Heat shock protein HSP90-1 |
| C-W-10 | tr\|Q2TFP3\|Q2TFP3 | PIP2 |
| C-W-11 | tr\|O49855\|O49855 | Acid phosphatase |
| C-W-12 | tr\|I1J582\|I1J582 | Eukaryotic translation initiation factor 3 subunit I |
| C-W-13 | tr\|C6TI33\|C6TI33 | Obg-like ATPase 1 |
| C-W-14 | tr\|Q2LAJ5\|Q2LAJ5 | Cytochrome P450 monooxygenase CYP74A1 |
| C-W-15 | tr\|I1KVD8\|I1KVD8 | Phospholipase |
| C-W-16 | tr\|A0A0R0ESZ8\|A0A0R0ESZ8 | Elongation factor 1-alpha |
| C-W-17 | tr\|A0A0R0J5A9\|A0A0R0J5A9 | Lipoxygenase |
| C-W-18 | tr\|C7S8D5\|C7S8D5 | Germin-like protein 1 |
| C-W-19 | tr\|I1KJL7\|I1KJL7 | Oxygen-evolving enhancer protein 3 |
